# Expired Patents

**Published:** 1906-09

**Authors:** 


### EXPIRED PATENTS.

July 30, 1906.

407,950, Stop motion for dental engines,A. R. Cooke, Syracuse, N. Y.

August 6, 1906.

408,439, Dental engine, L. T. Sheffield, New York, N. Y.

August 13, 1906.

408,739, Dental forceps, N. G. Nyblin, Stockholm, Sweden.

408,883, Dental plunger, S. Lacavalerie and C. Doriot, Phila., Pa.

408,898, Apparatus for allaying the sensibility of a tooth, D. A. Small, Providence, R. I.

408,923, Dental drill attachment, J. H. Moyer and J. P. Stansell, Temple, Tex.

408,926, Dental file, C. E. Palmer, New Haven, Conn.

The above list of patetns, are furnished by Davis & Davis, solicitors of American and foreign patents, Washington, D. C., and St. Paul Building, New York City.

